# Evaluation of a results-based financing nutrition intervention for tuberculosis patients in Madhya Pradesh, India, implemented during the COVID-19 pandemic

**DOI:** 10.1186/s44263-023-00013-6

**Published:** 2023-09-04

**Authors:** Embry Howell, Rama Rao Dammala, Pratibha Pandey, Darcy Strouse, Atul Sharma, Neeta Rao, Sudheer Nadipally, Amar Shah, Varsha Rai, Russell Dowling

**Affiliations:** 1 Urban Institute, 500 L’Enfant Plaza SW, Washington, DC 20024 USA; 2 ChildFund India, 22, Museum Road, Bengaluru, Karnataka 560001 India; 3 ChildFund International, 2821 Emerywood Parkway, Richmond, VA 23294 USA; 4 Postgraduate Institute of Medical Education and Research, Sector-12, Chandigarh, PIN- 160012 India; 5 US Agency for International Development, 1300 Pennsylvania Avenue NW, Washington, DC 20004 USA; 6 IPE Global, B-84, Defence Colony, Delhi, New Delhi 110024 India; 7 State Tuberculosis Office, National Health Mission, Link Road No. 3, Journalist Colony, Bhopal, PIN-462016 India

**Keywords:** Tuberculosis, Nutrition, Evaluation, India, Results-based financing

## Abstract

**Background:**

Reducing malnutrition through food supplementation is a critical component of the WHO End Tuberculosis (TB) strategy. A results-based financing (RBF) initiative in Madhya Pradesh, India—called Mukti—introduced an intensive nutrition intervention, including home visits, counseling, food basket distribution, and assistance in obtaining government benefits. Phase 1 of the program (Dhar District), implemented by ChildFund India (ChildFund) and funded by USAID, coincided with the COVID-19 lockdown in 2020. Under an RBF reimbursement scheme, ChildFund was paid based on treatment retention for 6 months and weight gain of 6 kg for adults.

**Methods:**

The evaluation used a mixed methods approach. Qualitative components included interviews with key informants and focus groups with program participants. Quantitative components included an analysis of program data (i.e., patient demographics, receipt of program services, and weight gain). An impact analysis of retention in treatment used data from a government database. A difference-in-differences model was used to compare results from baseline data and the program period for Dhar District to similar data for the adjacent Jhabua District.

**Results:**

The program was well implemented and appreciated by patients and providers. Patients received an average of 10.2 home visits and 6.2 food baskets. While all age and sex groups gained weight significantly over their 6-month treatment period, there was no program impact on treatment retention. Seventy-six percent of patients achieved both outcome goals. And though average program costs were under budget, ChildFund experienced a loss in the results-based financing scheme, which was covered by USAID to continue program expansion.

**Conclusions:**

Implementing a nutrition supplementation and education program for TB patients in India is feasible. The intervention improved weight gain despite COVID-19-related lockdowns. The Mukti program did not impact treatment retention, which was already high at baseline. Program costs were modest, but the results-based financing reimbursement scheme resulted in a loss for the implementer. Overall, the RBF model led to an increased focus on outcomes for program staff and other stakeholders, which led to more efficient service delivery. Future research should examine total costs (including donated staff time) more extensively to determine the cost-effectiveness of Mukti and similar interventions.

**Supplementary Information:**

The online version contains supplementary material available at 10.1186/s44263-023-00013-6.

## Background

Tuberculosis (TB) is an infectious disease caused by the bacillus Mycobacterium tuberculosis, which is spread when people who are sick expel bacteria into the air (e.g., by coughing). About a quarter of the global population is estimated to have been infected with TB, although most people will never develop active TB. While progress has been made toward controlling TB in recent decades, progress towards eliminating TB has stalled worldwide. Achievement of a much more ambitious goal, the “End TB” strategy to eliminate TB entirely by 2035, is now in question. Even before the onset of the COVID-19 pandemic, data indicated that the global cumulative rate of reduction of TB incidence would likely be below the milestone of 20% reduction between 2015 and 2020 [1–3].

Two closely intertwined factors have been identified as being prominently associated with TB disease burden: poverty and poor nutrition. Poverty increases the risk of TB through poor nutrition, poor quality or informal housing (e.g., with poor ventilation), and risky health behaviors such as smoking [4]. Using data from various sources, researchers estimate that ending extreme poverty would result in a 33.4% reduction in TB incidence by 2025 [5]. Malnutrition affects the immune system, and increases the activation of latent TB, among other adverse patient outcomes [6, 7]. Further, research shows that mortality from TB decreases as malnutrition decreases, both at the individual and population levels [8, 9]. Reducing malnutrition through food supplementation is viewed as a critical component of the End TB strategy [10]. However, research on the impact of supplementation on mortality and weight gain has shown mixed results according to a recent Cochrane systematic review [11].

Rates of poverty and malnutrition have been greatly impacted by the COVID-19 pandemic [12]. Although not yet closely studied, the worldwide food shortage linked to COVID is also very likely increasing TB incidence. One study showed that TB case notifications were down worldwide in 2020, and that India contributed 41% to the global decline in TB case notification [13].

Many India-specific studies have confirmed the close relationship between poverty, nutrition, and TB [14–18]. Addressing TB in India must go hand-in-hand with addressing poverty and associated hunger [14, 19]. One study demonstrated that the odds of having TB among people living below the poverty threshold in India was about twice that of people living above the threshold [20]. Further, another study concluded that over half of all active TB cases in India were attributable to malnutrition, most prominently in rural areas and among scheduled castes and tribes [21], as well as in the central and western regions of India [22]. TB recurrence and case fatality are also high in India [23, 24], with one study showing that only 37% of patients experience recurrence-free survival in contrast to 53% in South Africa [25]. According to other estimates, reductions in malnutrition could avert up to 71% of India’s TB deaths [26, 27]. However, three India-specific studies of the impact of nutritional supplementation found mixed results [28–30]. As also is true worldwide, the COVID-19 pandemic exacerbated poverty and food insecurity in India, with one study estimating a 20% increase in deaths from TB associated with COVID [31].

The India National Strategic Plan for TB for 2017–2025 proposed putting into place several new initiatives to work towards eliminating TB. Recognizing the critical role of nutrition support, one of the new initiatives is nutritional support through Direct Benefits Transfer (DBT) to patient bank accounts [32, 33]. Each TB patient is to receive 500 rupees (approximately USD 6.50) a month to pay for nutrient-dense food. In the year after DBT was implemented, three different studies in various parts of India concluded that the system was not yet providing subsidies to most patients due to administrative delays, lack of patient bank accounts, and lack of training of TB program staff on the new system. Those who did receive support had to wait several months, limiting the utility of the intervention [34–36]. However, these studies were completed soon after implementation; the reach and efficiency of the program may have improved substantially since that time. Furthermore, the subsidy amount of 500 rupees is only a quarter of the amount of a typical monthly food basket, estimated at 2,000 rupees, for a poor Indian family in 2022 [37].

One means of improving the quality, efficiency, and transparency of health and social programs is to pay programs or providers based on their results. While the main goal of these Results-Based Financing (RBF) programs is to improve outcomes, another goal is to reduce cost through improved efficiency.

RBF programs gained traction in the early 2000s in the USA and Europe, whereby physicians or hospitals were paid bonuses (or even risked reduced payments) according to quality-of-care scores, with mixed results [38, 39]. Another approach is to use “impact bonds”, which have a more complex structure [40]. As with other RBF programs, the results from impact bonds have been mixed [41, 42]. A Cochrane systematic review of RBF programs in low- and middle-income countries found some improvements in a variety of outcomes, especially for maternal and child health programs [43]. RBF approaches have expanded worldwide, including to many low- and middle-income countries, in spite of mixed results from evaluations [44–46]. One reason for mixed results is the incredible diversity of approaches and types of programs that fall under the umbrella of RBF [47]. In addition, the initial effects of small, pilot programs may be difficult to scale-up successfully [48].

Several RBF programs in India have been evaluated; most are maternal and child health programs. Only one of the studies, from Bihar, used a randomized design, and it was a relatively small study with 300 patients each in the treatment and control groups. It found that team-based incentives increased the frequency of antenatal home visits and receipt of iron-folic acid tablets [49]. However, the evaluation of an impact bond to improve maternal health quality and outcomes found no significant impact on outcomes at midline [50]. Several other evaluations did find significant improvements, although they had limitations in their designs, particularly a lack of an adequate comparison group. In Jodhpur, implementing performance bonuses for community health workers was associated with increases in services [51]; bonuses to daycare center workers in Chandigarh led to improved weight-for-age among children of the centers [52]; and Karnataka health providers serving pregnant women and children who were paid per patient increased institutional delivery and immunization rates [53]. In contrast to these positive results, a program in Mumbai providing incentives to increase peer mobilizers’ referrals to HIV treatment led to few referrals due to stigma [54].

There are few findings from RBF TB programs reported in the literature (and none in India). One program in Pakistan provided incentives to family planning clinics to identify smear-positive TB cases and refer them to treatment. This led to increased referrals to TB treatment for those seeking care in the private sector, although it increased overall costs [55]. Two studies in Taiwan also examined TB outcomes from RBF programs. In the first study, providers volunteered to receive an incentive ($183) to treat TB patients. Patients of those providers were more likely to complete treatment and cost $215 (4.6%) less than patients of providers who did not volunteer [56]. The other Taiwanese study compared outcomes pre- and post-implementation of RBF for hospitals treating TB patients. The cure rate in RBF hospitals increased from 46.9% before RBF to 63.0% after implementation [57].

## Methods

### The Mukti program

**Fig. 1:**
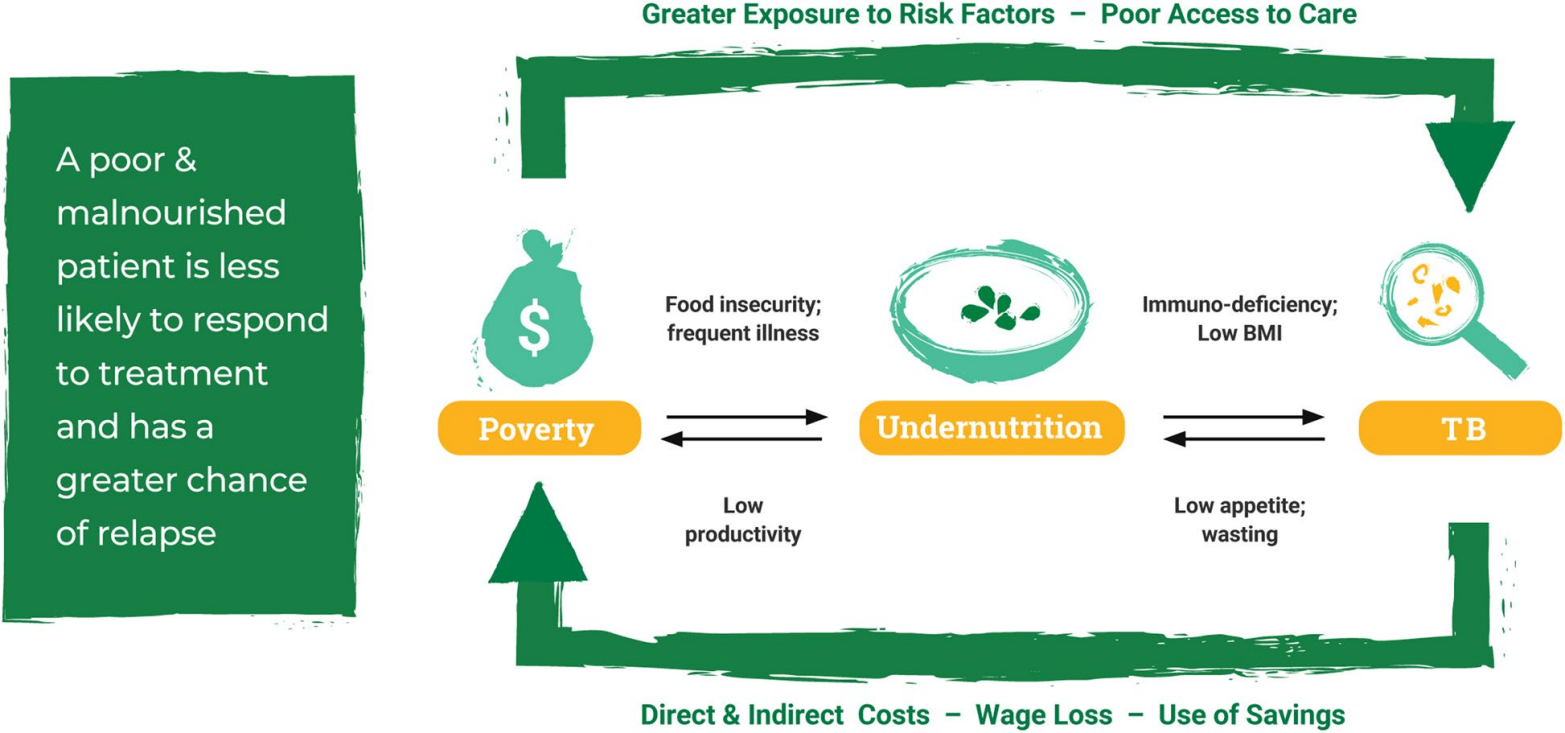
Relationship between nutrition and TB

Recognizing the acute need for nutritional support for TB patients, and building on its experience delivering high-quality nutrition interventions, ChildFund India, in partnership with the United States Agency for International Development (USAID) and the state TB program under the Government of Madhya Pradesh, set out in early 2018 to design and implement a results-based financing approach to improve the nutritional status of its TB patients [58]. The program designers used guidance from WHO India concerning nutrition for TB patients [59]. Simultaneously, USAID embarked on an effort in India to use innovative financing approaches for its grant programs through a partnership with IPE Global—an international development consultancy group that provides technical assistance on development projects. The IPE Global project is called Partnerships for Affordable Healthcare Access and Longevity (PAHAL) [60]. After lengthy discussions and a preparatory pilot project, a concept was developed for a results-based financing program called “Mukti,” meaning “delivery from harm.” An overview of the underlying motivation for the Mukti program design is illustrated in Fig. 1.

Madhya Pradesh has a total population of over 72 million across 52 districts, with a relatively high rate of poverty according to various indicators compared to national rates. For example, in Madhya Pradesh only 65.1% of households live in housing with improved sanitation (compared to 70.2% nationally). The initial district for implementing Mukti was Dhar District in Madhya Pradesh. The average resident of Dhar District is poorer than the average resident of Madhya Pradesh (55.6% female literacy and 61.9% of households with improved sanitation). Dhar also has a very high proportion of vulnerable tribal residents [61].

Informed by the pilot results, in collaboration with state and district TB administrators, ChildFund India launched Mukti in Dhar District, with a goal of initially serving 1000 TB patients (called “phase 1”) from all 31 Designated Microscopy Centers (DMCs). Mukti adopted an RBF model whereby USAID would pay ChildFund India based on two indicators of success: treatment completion and weight gain of more than 6 kg. The financial incentive was given to ChildFund (rather, for example, than to the treatment facilities), because of its experience with nutritional supplementation and because it could rapidly hire program staff. The phase 1 program designers (USAID, IPE Global, the Madhya Pradesh government, and ChildFund India) developed a per-patient budget, which was used to estimate the reimbursement that would be obtained for each phase 1 patient that achieved both outcome targets. The agreed upon per patient payment was $166, based on the costs of the program during the pilot period. The concept was that—should Mukti cost less per-patient than the $166 per-patient estimate—ChildFund would obtain a “profit” that could be used for program expansion. Program designers also assumed that Mukti would be expanded to other districts within Madhya Pradesh, and that overhead costs would go down. The Dhar District government, with guidance from the state TB officer, was a key partner as the entity that measured treatment success and certified that ChildFund had met its targets.

The phase 1 program design included five interrelated activities, all designed to improve treatment compliance and nutritional status. These included (1) one-on-one counseling during home visits to assure TB treatment compliance; (2) one-on-one nutrition counseling during home visits; (3) group nutrition counseling during “positive deviance” (PD) group counseling sessions, held during food-basket distribution visits; (4) help in obtaining government Direct Benefit Transfer benefits; and (5) delivery of nutrient-dense food baskets. The content of food baskets varied according to the patient’s nutritional needs, but the standard food basket content is profiled in Table 1. ChildFund India hired 15 new employees, called “cluster coordinators,” who delivered all project services, including home visits, PD sessions, and food basket distribution. The service area was divided into “blocks” of 100,000–200,000 people, with each cluster coordinator responsible for two blocks. Cluster coordinators were responsible for enrolling all TB patients in the allotted time period into this study.

**Table 1:** Food basket components

**For adults**				
**Item**	**Quantity (kg)**	**Per day (g)**	**Calories (kcal/day)**	**Protein (g/day)**
**Atta/wheat flour**	4	133	465	11
**Ground nut**	2	65	360	16
**Toor dal**	1	35	122	7
**Sattu (roasted chana and wheat)**	1	35	142	7
**Total**	8 kg	268 g/day	1089 kcal	41 g/day
**For children**				
**Item**	**Quantity (kg)**	**Per day (g)**	**Calories (kcal/day)**	**Protein (g/day)**
**Atta/wheat flour**	3	100	350	8
**Ground nut**	0.5	17	96	4.5
**Toor dal**	1	35	122	7
**Sattu (roasted chana and wheat)**	0.5	317	71	3.5
**Total**	5 kg	169 g/day	639 kcal	23 g/day

Mukti phase 1 was launched just as the COVID-19 nationwide lockdown began, with the first patient enrolled in Mukti on 16 March 2020. Home visits were allowed from the beginning of the program as an exception for health workers. However, provision of the full package of program services for all patients was not achieved until May 2020. The program continued enrolling patients through the end of September 2020 and continued following those patients through treatment completion in April 2021.

### The Mukti evaluation

This paper provides results from an evaluation of phase 1 of the Mukti program. Evaluation objectives and associated research questions are shown in Table 2.

**Table 2:** Evaluation objectives and research questions

Objective	Research questions
1—Examine the implementation of the program and patient impressions of the program	• What services were provided to Mukti patients?
	• How did those services vary over time and by type of patient?
	• What were the impressions of key informants about the Mukti program services?
2—Assess the effect of Mukti on the targeted outcomes of weight gain and treatment completion	• Did Mukti patients gain weight, and how did weight gain vary by type of patient and number of services received?
	• Did Mukti improve treatment completion in Dhar District when compared to Jhabua District where there were no Mukti services during the time period?
3—Examine program costs and reimbursement for those costs	• What were the costs of the intervention?
	• Did the results-based financing design affect the outcomes of the project?
	• Did the project cover full costs through the results-based payments?

### Evaluation design

**Fig. 2:**
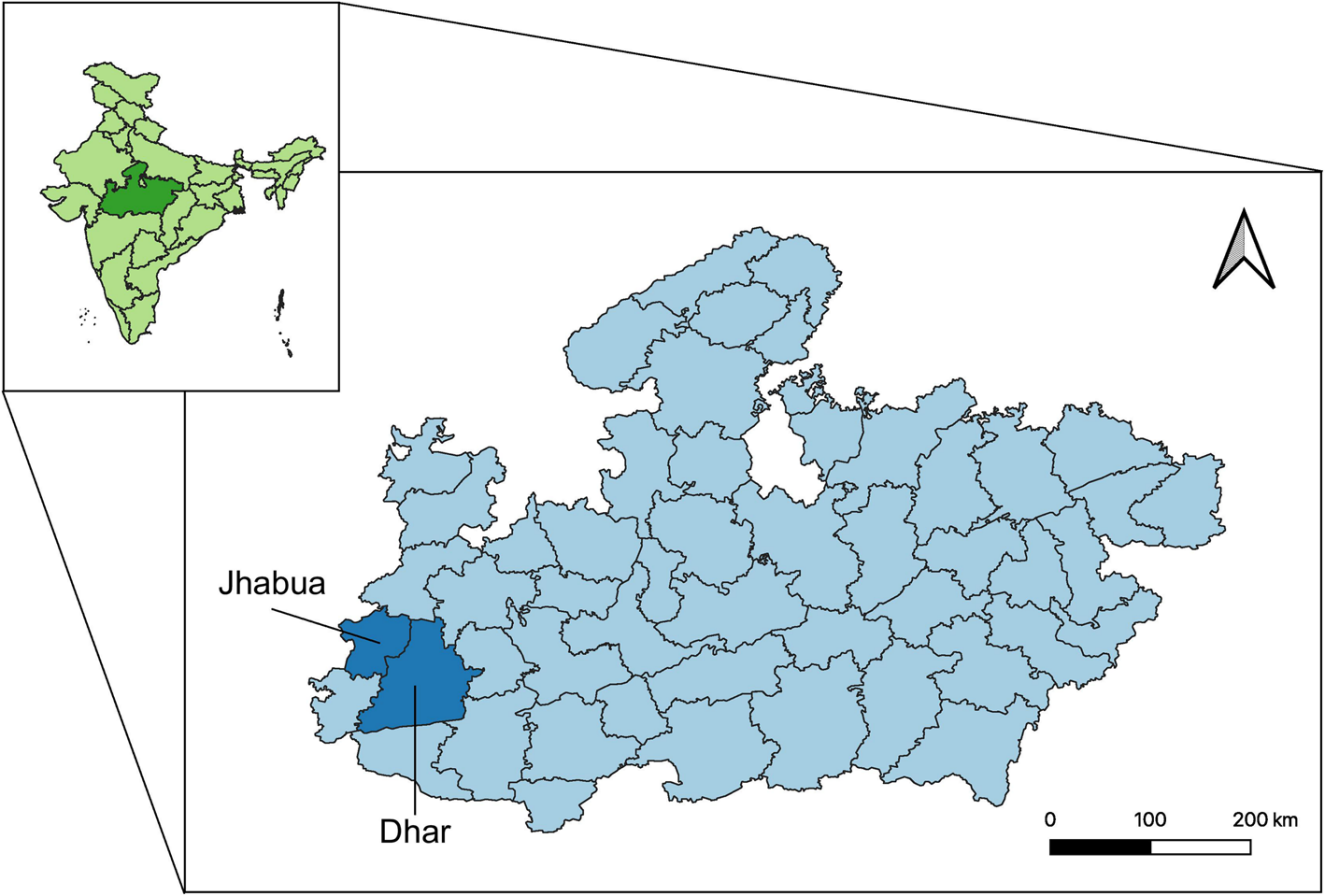
Map of Madhya Pradesh: location of the intervention district, Dhar, and the comparison district, Jhabua

The evaluation used a mixed methods approach, with both qualitative and quantitative information, to address the evaluation objectives and examine the implementation, outcomes, and cost of Mukti phase 1. The effects of the program on the two major programmatic outcome indicators were examined in two ways. For weight gain, we examined the change in weight among Mukti participants from baseline to treatment completion, or when the patient dropped out or died. For treatment completion, we used a quasi-experimental difference-in-differences examination of the program’s impact. For this latter analysis, changes in treatment completion rates before and after Mukti in Dhar District were compared to changes in the same time period in adjacent Jhabua District. Jhabua District has similar socio-demographics to Dhar District and had not yet started Mukti programming during the study time period. For example, Jhabua female literacy was only 37.1% and only 57.6% of households had improved sanitation at the time of the study. Figure 2 shows the locations of Dhar and Jhabua districts within Madhya Pradesh.

### Data sources

#### Qualitative

Key Informant Interviews (KIIs) were used to collect general project impressions from USAID staff, ChildFund current and former staff, IPE Global staff, and two Medical Officers for TB treatment facilities in Dhar District (Additional file 1). In addition, the Postgraduate Institute of Medical Education and Research (PGIMER), Chandigarh, India, conducted three focus group discussions (FGDs) in Hindi with 18 Mukti patients and their family members in three randomly selected DMCs (Additional file 1). Patients were recruited through text messages and by telephone. Moderators led the discussions using a semi-structured discussion guide and used open-ended questions to prompt free discussion.

#### Quantitative

The source of quantitative data on Mukti participant characteristics, services obtained by Mukti program participants, and Mukti program cost was the program data maintained by ChildFund India. Weight gain data were collected and recorded by cluster coordinators using portable scales each time they provided other project services.

The second quantitative data source is the database maintained throughout India for TB services provided in government facilities, called “Nikshay” [62]. Nikshay was used as the source of treatment completion data for both Dhar and Jhabua districts. All government-funded TB treatment facilities (DMCs) are required to participate in Nikshay and enter a record of each patient who begins treatment and a record for each patient who completes treatment, along with patient demographic characteristics and other variables. We obtained one summary record per month for each age group and each sex group for all patients initiating TB treatment and all patients completing TB treatment in each district, for each month in the time period May 2018 through February 2021. Individual-level data could not be obtained due to confidentiality restrictions.

### Data analysis

#### Qualitative

Qualitative data from the FGDs were digitally recorded, transcribed verbatim, translated into English, and analyzed manually to formulate themes. The KII interview notes and FGD transcripts were analyzed by the same qualitative researcher, and results were combined for an overall qualitative assessment of project impressions.

#### Quantitative

Tabular data on program implementation, patient demographics, and weight gain were analyzed using SPSS version 26. Paired *t* tests were used to test the significance of weight gain changes.

For the analysis of the program’s impact on treatment completion, the monthly aggregate data from Nikshay were used to calculate a treatment “completion ratio” for each month beginning in November 2018 through February 2021. The completion ratio is the number of people completing treatment in the month divided by the number of people initiating treatment 7 months earlier, given that the treatment period is generally 6 months. Completion ratios were calculated for each month and for six age/sex cohorts: females < 21 years; females 21–40; females 41 + ; males < 21 years; males 21–40; males 41 + , for both districts (Dhar and Jhabua), and for 28 months. This resulted in 336 monthly summary records. Children under 2 were excluded from the program and the analysis, as were pregnant women.

To facilitate a pre/post intervention comparison of treatment completion—and also an examination of the effect of COVID-19 (and other secular trends) on treatment completion—the treatment completion data were further divided into five discrete time periods that covered both pre-phase 1 and phase 1 Mukti time periods. Additional file 1 provides a detailed explanation of the time periods. Two critical time periods for the analysis are:

- *A comparison period* in which patients received no Mukti programming from treatment initiation to completion; this includes 3 months of completion ratios for November 2019–February 2020.
- *A study period* in which patients received full Mukti programming from treatment initiation to completion; this includes 4 months of completion ratios for November 2020–February 2021.

The other three time periods were used to control for secular trends affecting both districts; each of the three periods had some minor Mukti programming as explained in Additional file 1.

To assess program impact on treatment completion, a Difference-in-Differences model using linear regression analysis was conducted with SPSS version 28. The completion ratio was the outcome variable, and control variables were age, sex, district, and time period. The impact of Mukti is the interaction between Dhar District and the study time period (a “Difference-in-Differences” design), as follows:

$$\mathrm{Y }=\mathrm{ a }+\mathrm{ b}(1)\mathrm{X}(1)+\mathrm{b}(2)\mathrm{X}(2)+\mathrm{b}(3)\mathrm{X}(3)+\mathrm{b}(4)\mathrm{X}(4)+\mathrm{b}(5)\mathrm{X}(5)$$

Where: X(1) = age.

X(2) = sex.

X(3) = district.

X(4) = time period.

X(5) = interaction between study period and district.

## Results

### Program implementation

During the study period (Mukti phase 1) 1000 TB patients enrolled in Mukti, from all Dhar DMCs, which was the number that could be accommodated using the allotted project budget. Table 3 shows the demographic characteristics of the phase 1 Mukti patients at the time of enrollment in the program.

**Table 3:** Demographic characteristics of Mukti phase 1 patients

Age (years)	*n*	%
2–10	84	8.4%
11–20	159	15.9%
21–30	304	30.4%
31–40	206	20.6%
41–50	126	12.6%
51–60	76	7.6%
60 +	45	4.5%
Sex	*n*	%
Male	590	59%
Female	410	41%
Total	1000	100%

**Fig. 3:**
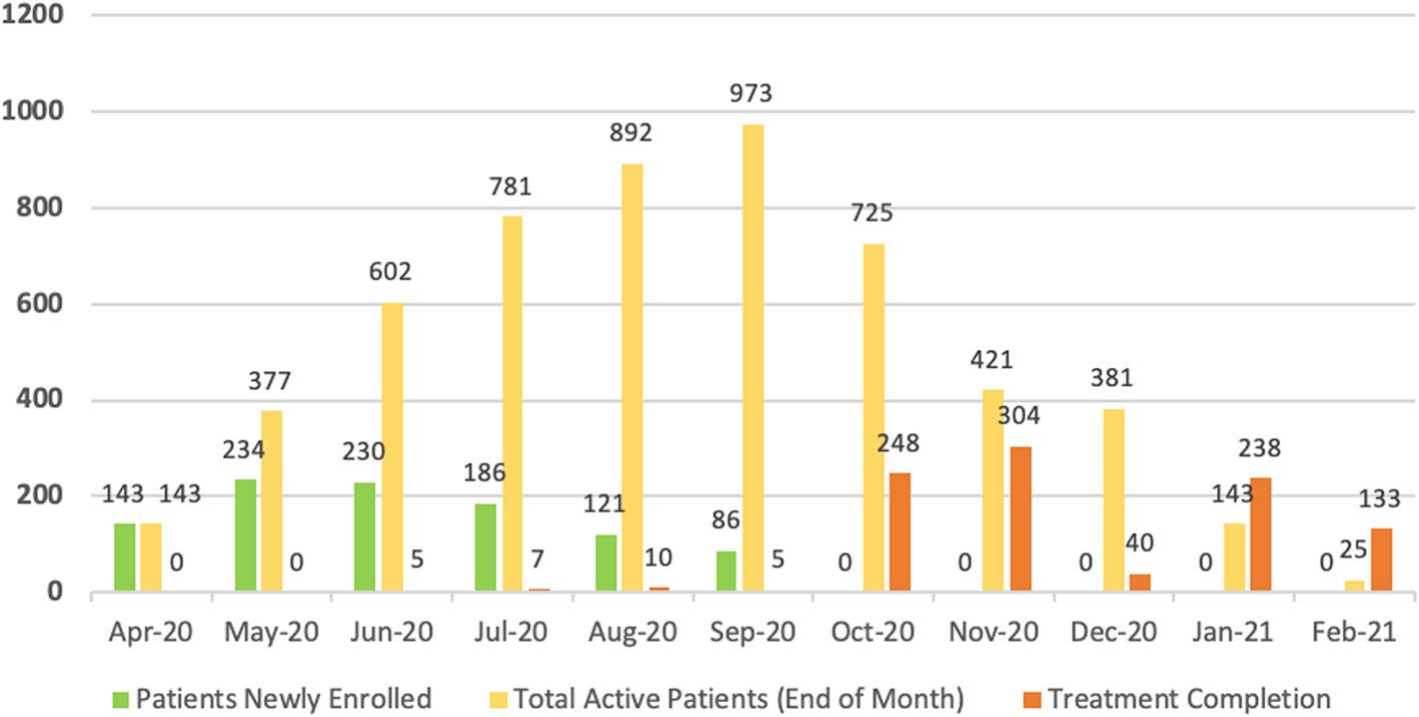
Number of Mukti patients by treatment status: April 2020 to February 2021

Mukti activity began somewhat slowly in April, 2020 (due to COVID-19), but quickly accelerated throughout May and June. By mid-September, 2020, Mukti had enrolled the full targeted number of 1000 patients. During that time period, 43 patients either dropped out or died, meaning the total number of active patients peaked in September 2020 at 973 patients. Since the prescribed treatment period for pulmonary TB was 6 months, by September, patients began leaving the program when they completed treatment. The final patients were served by the program in February, 2021 (with 5 multi-drug resistant or MDR TB patients who continued for two more months). Figure 3 shows the monthly total of phase 1 Mukti patients, both newly enrolled and active during the time period April 2020 through February 2021.

### Program impressions

Program feedback from both key stakeholders and program participants revealed that the Mukti program was perceived as being well implemented and beneficial—both in the delivery of specific services and the yielding of specific outcomes. In particular, key informants, such as the Medical Officers in charge of the DMCs, stated that they appreciated the efforts of the Mukti cluster coordinators. They felt that the home visits, food baskets, and PD sessions made a large difference in both weight gain and treatment completion. They remarked that they noticed a reduction in treatment success during the down period between the pilot time period and phase 1 of Mukti. One respondent remarked that they were impressed that the cluster coordinators were “well-managed and effective.” Another remarked that Mukti home visits were especially important during the early period of the COVID-19 lockdown when some treatment facilities were closed, since the cluster coordinators could deliver drugs to the patients during home visits.

The focus groups confirmed that patients were grateful for Mukti services. Patients reported that the most common services from the cluster coordinators were treatment adherence counseling and monitoring; nutrition counseling (including encouragement to consume the food provided in the food baskets); and weight gain measurement. They also appreciated services and the caring approach of their cluster coordinators, as one patient shared:

“*They talk very lovingly; they come again and again and ask about my well-being.”*

Most patients recounted receiving 5 or 6 food baskets. Some patients said that they often shared their food with their families and ran out of food mid-month:

“*It gets over in 10 to 12 days. If I eat it alone, it can last 15 days.”*

In reporting on the positive deviance sessions, patients indicated that the sessions lasted 45–60 min, in which the cluster coordinators talked about the importance of nutritious food, and especially locally available items. As recalled by one patient:

“Everyone tells us what should be eaten, how to make it, and from where to get it. They tell us about the benefits of good food.”

Since food baskets were distributed at the positive deviance sessions conducted at DMCs, many patients admitted attending these sessions primarily as a means to receive their food basket. Patients shared that they reverted to traditional eating habits after treatment ended, when they no longer received Mukti home visits, food baskets, and PD sessions. According to one patient:

“There was a lot of emphasis on fruits and vegetables at the time of treatment; now we eat as we did before.”

Help with DBT payments was especially important in rural areas; residents of urban areas did not appear to rely on the DBT payment to the same extent. For example, a patient from a rural area shared:

“It is very important. It is useful in covering household expenses.”

### Program services

Across the time period of the intervention, a total of 10,723 home visits were conducted (an average of 10.7 per patient ever active in the program). Additionally, patients received counseling at 431 PD sessions. Since an average of 5 patients attended each session, patients each attended an average of 2.2 PD sessions. Finally, 6184 nutritious food baskets were distributed during the program, an average of 6.2 per Mukti patient. The averages for home visits, PD sessions, and food baskets did not vary significantly by age or sex. As planned, the median number of food baskets was 6 (about one per month during treatment). The number of food baskets delivered was between 1 and 8. The median number of PD sessions was close to the target of 2.

The intensity of home visit services varied by month, with the average patient receiving more home visit services later in the program. Most of the 15 cluster coordinators were kept on board through December, 2020, and thus they were able to do more home visits per active patient. PD sessions were concentrated in the months of August-November 2020. In addition to these services, Mukti patients were assisted in receiving Direct Benefits Transfers to their bank accounts from the government’s DBT system. Fully, 96.4% of Mukti patients received DBT benefits.

### Program outcomes

#### Weight gain outcomes

Table 4 shows weight gain by demographic characteristics for adults. The target adult weight gain for the program was that each adult would gain at least 6 kg during their treatment. On average, all sex and age groups met the 6 kg target for adults, except for men ages 50 + , and women ages 31–40 and 50 + , with women aged 31–40 showing the smallest weight gain (4.8). However, all 8 demographic groups yielded a significant change (*p* < 0.01) in mean weight.

**Table 4:** Adult weight gain by age and sex

Demographic characteristics	Number of patients	Mean length of treatment (days)	Mean weight at start of treatment (kg)	Mean weight at end of treatment (kg)	Mean change in weight (kg)
Males					
Ages 21–30	165	232	44.0	50.1	6.0*
Ages 31–40	138	232	45.4	51.2	5.7*
Ages 41–50	85	238	44.7	50.8	6.1*
Ages 50 +	87	216	45.0	50.0	5.0*
Females					
Ages 21–30	139	236	38.8	44.6	5.8*
Ages 31–40	67	233	40.1	44.9	4.8*
Ages 41–50	41	243	42.6	48.2	5.6*
Ages 50 +	33	227	40.4	45.4	5.0*
Total adults	755	233	42.7	48.4	5.7*

^*^Change significant, *p* < 0.01

**Fig. 4:**
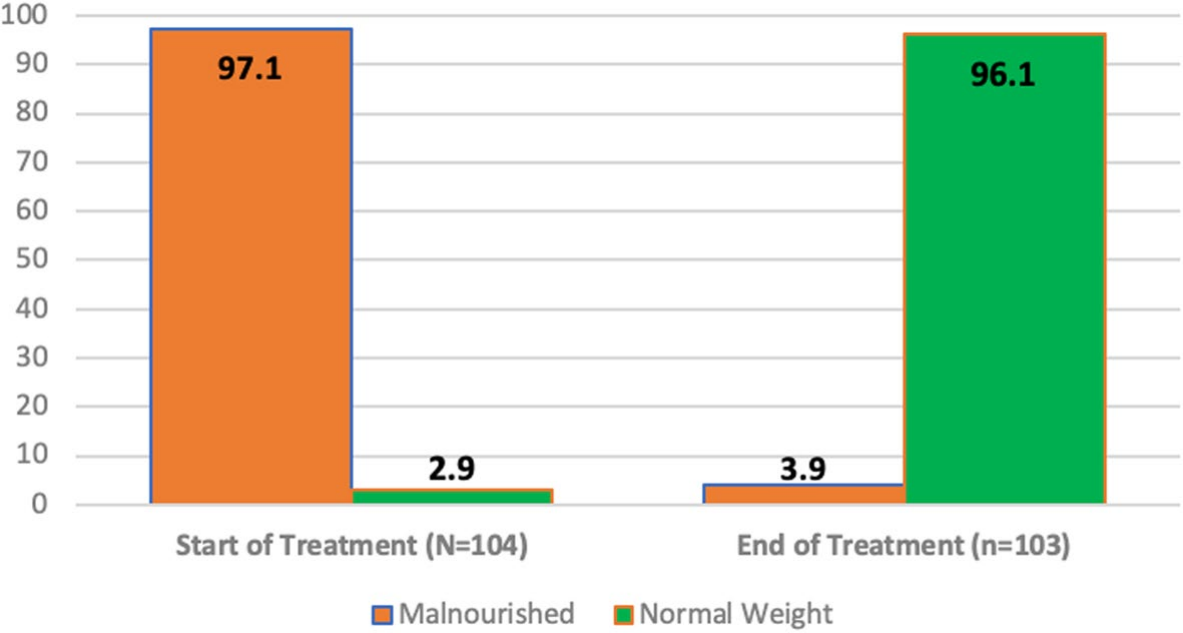
Weight gain for children. Notes: (1) Malnourished is defined as less than 1 standard deviation below the norm for the WHO *z* score for age; and (2) Ns for start (*N* = 104) and end of treatment (*N* = 103) are different due to 1 death during the project period

Children’s weight was evaluated according to the WHO *Z*-score standards. In order to meet the Mukti target for weight gain, at the end of treatment, children were expected to be within one standard deviation of the WHO standard for normal weight for a child’s age. Weight gain among children in Mukti is displayed in Fig. 4. For children, there was an even more dramatic Mukti effect on weight gain. While almost all children (97.1%) were malnourished prior to Mukti, fully 96.1% (99 of the remaining 103 children) were within the normal range of weight for their age according to WHO standards after treatment and Mukti services.

While there is no information on weight gain for a non-Mukti cohort during the study period, one source of comparison data was collected during the Mukti pilot phase, when 195 patients were studied in DMCs in Dhar District where Mukti was not offered [63]. A large percentage (70.5%) of those patients gained less than 4 kg during treatment. This was also confirmed by qualitative information from treatment providers who stated that their patients’ outcomes improved under Mukti. Another study is underway during phase 2 of Mukti that will collect data on weight gain for a comparison group.

As shown in Tables 5 and 6, a relationship is observed between weight gain and the receipt of food baskets and home visits, respectively. Although we see significant change in weight gain for patients receiving either 3–4 and 5–6 food baskets (Table 5), patients who had more than six food baskets per month were the most likely to meet the target of 6 kg weight gain. Those receiving fewer baskets generally included patients who did not have a full 6 months of treatment, so for those patients the period over which weight gain was measured was shortened. (The average weight gain during the first 2 months was 2 kg—data not shown.) It is notable that those with 7–8 food baskets had the highest weight gain of all groups, including those with 9 + baskets.

**Table 5:** Weight gain by number of food baskets

Number of food baskets	Number of patients	Mean length of treatment (days)	Mean weight at start of treatment (kg)	Mean weight at end of treatment (kg)	Mean change in weight (kg)
0–2	15	31	34.2	34.4	0.2
3–4	131	33	39.7	43.3	3.6*
5–6	610	239	39.8	45.4	5.6*
7–8	242	248	38.9	44.9	6.0*
Total	998	226	39.4	45.6	5.4*

^*^Change significant, *p* < 0.01

**Table 6:** Weight gain by number of home visits

Number of home visits	Number of patients	Mean length of treatment (days)	Mean weight at start of treatment (kg)	Mean weight at end of treatment (kg)	Mean change in weight (kg)
0–2	9	31	37.1	37.1	0.0
3–4	17	29	35.2	36.4	1.1
5–6	35	133	36.1	40.3	4.2*
7–8	97	204	37.8	43.6	5.8*
9 +	840	248	39.9	45.4	5.5*
Total	998	226	39.4	45.6	5.4*

^*^Change significant, *p* < 0.01

A similar pattern is seen concerning weight gain according to the number of home visits received (Table 6). The weight gain target was closest to being achieved, on average, for those receiving seven or more home visits. Because home visits have a dual purpose, counseling for treatment completion and nutrition counseling, there may be a double effect of the visits, since patients who are treatment compliant may also gain more weight.

#### Treatment completion

Table 7 shows the number of TB patients tracked in Nikshay entering and completing treatment for each district, age, sex, and time period, as well as the aggregate completion ratios for each category of patients by district.

**Table 7:** TB treatment completion ratios by district, demographic characteristics, and time period

	Dhar district			Jhabua district		
Demographic category	Number (%) entering treatment	Number completing treatment	Completion ratio	Number (%) entering treatment	Number completing treatment	Completion ratio
Males ages 3–20	1591 (15.7)	1503	0.94	476 (9.2)	420	0.88
Males ages 21–40	2641 (26.1)	2296	0.86	1358 (26.2)	1111	0.82
Males ages 41 +	2282 (22.5)	1921	0.84	1668 (32.2)	1443	0.87
Females ages 3–20	1209 (11.9)	1215	1.01	411 (7.9)	365	0.89
Females ages 21–40	1711 (16.9)	1578	0.92	829 (16.0)	760	0.92
Females ages 41 +	697 (6.9)	660	0.95	432 (8.3)	450	1.04
**Time periods**						
Baseline period 1 (13 months)	5523	5197	0.94	2420	2324	0.96
*Comparison period (3 months)*	*1083*	*1072*	*0.99*	*570*	*484*	*0.85*
Baseline period 2 (5 months)	1769	1080	0.61	1148	638	0.56
Baseline period 3 (3 months)	869	635	0.73	418	412	0.99
***Study period (4 months)***	*887*	*1189*	*1.34*	*618*	*691*	*1.12*
**Total (28 months)**	10,131	9173	0.91	5174	4549	0.88

For the comparative analysis of treatment completion, 887 patients constitute the number of patients in the “Study Period” for this analysis. These patients represent the number of patients entering TB treatment in May–August 2020 and completing TB treatment in November 2020-February 2021—the treatment group for the full package of Mukti services. The number of patients actually entering the Mukti program during the time period was only 771 (a difference of 116 patients). Interviews with treatment providers revealed that not all patients were referred to Mukti, for various reasons such as administrative delays. Thus, this analysis is an “intent to treat” analysis, and the outcomes represent those who received Mukti services—86.9% of those in the analysis—and a much smaller percentage (13.1%) who did not receive Mukti.

As shown in Table 7, there were proportionately more children in Dhar than in Jhabua, and the age/sex distribution of TB patients in Dhar and Jhabua was significantly different. This difference is controlled for in the treatment completion analysis.

Across all time periods, the overall average completion ratio was 0.91 for Dhar District and 0.88 for Jhabua District. The completion ratio over 1.00 for the study period may be explained by the fact that patients who entered treatment during the earliest phase of COVID (February–March, 2020) may have had a delay in treatment and caught up during the study period.

Results from a regression analysis of aggregate monthly data on completion ratios, controlling for district, age, sex, and time period (see Table 8), revealed that age, sex, and time period were all significant predictors of treatment completion (at the *p* = 0.05 level or lower). Patients older than 40 years had significantly higher completion ratios and males had significantly lower completion ratios than females. Treatment completion ratios were also significantly lower during baseline time period three. These patients would have been scheduled to complete treatment during the early months of the COVID-19 pandemic (March 2020–July 2020). Thus, the lower completion ratio in that period may be attributed to the COVID-19 lockdown. During this time period, the completion ratio was only 0.61 in Dhar and 0.58 in Jhabua (see Table 8).

**Table 8:** Ordinary least squares regression predicting completion ratio

Variable	Coefficient (std. error)	*P* value
Constant	1.03 (0.14)	< 0.001
Dhar District	0.02 (0.08)	0.86
Male sex	− 0.17 (0.08)	0.03*
Age (omitted variable: age 21–40)		
Age 3–20	0.11 (0.09)	0.24
Age 41 +	0.20 (0.09)	0.04*
Time period (omitted variable: time period two/comparison period)		
Baseline time period 1	0.05 (0.13)	0.69
Baseline time period 3	− 0.38 (0.15)	0.01**
Baseline time period 4	0.29 (0.17)	0.08
Study period	0.17 (0.19)	0.36
Interaction of Dhar District and time period 5 (*impact measure*)	0.25 (0.22)	0.25

*Note*: *p < 0.05 level; **p < 0.01 level. *R* = 0.34; *R*^2^ = 0.12. Adjusted *R*^2^ = 0.09

The interaction term (study period/Dhar District), which reflected being treated for TB in Dhar with the full Mukti program versus not receiving the program services (in Jhabua), was not a significant predictor of treatment completion (coefficient 0.25, standard deviation 0.22, *p* = 0.25). After controlling for demographic factors in the model (age and sex), the two districts were almost identical in terms of completion ratios. It is possible that the effect of Mukti is dampened by the inclusion of some patients who did not actually receive any Mukti programming, as outlined above. In addition, 143 patients entered Mukti during March 2020, the COVID-19 lockdown period (see Fig. 3). These patients are included for this analysis in time period four. As shown in Table 8, time period four has an even higher coefficient (0.29, *p* = 0.08).

**Fig. 5:**
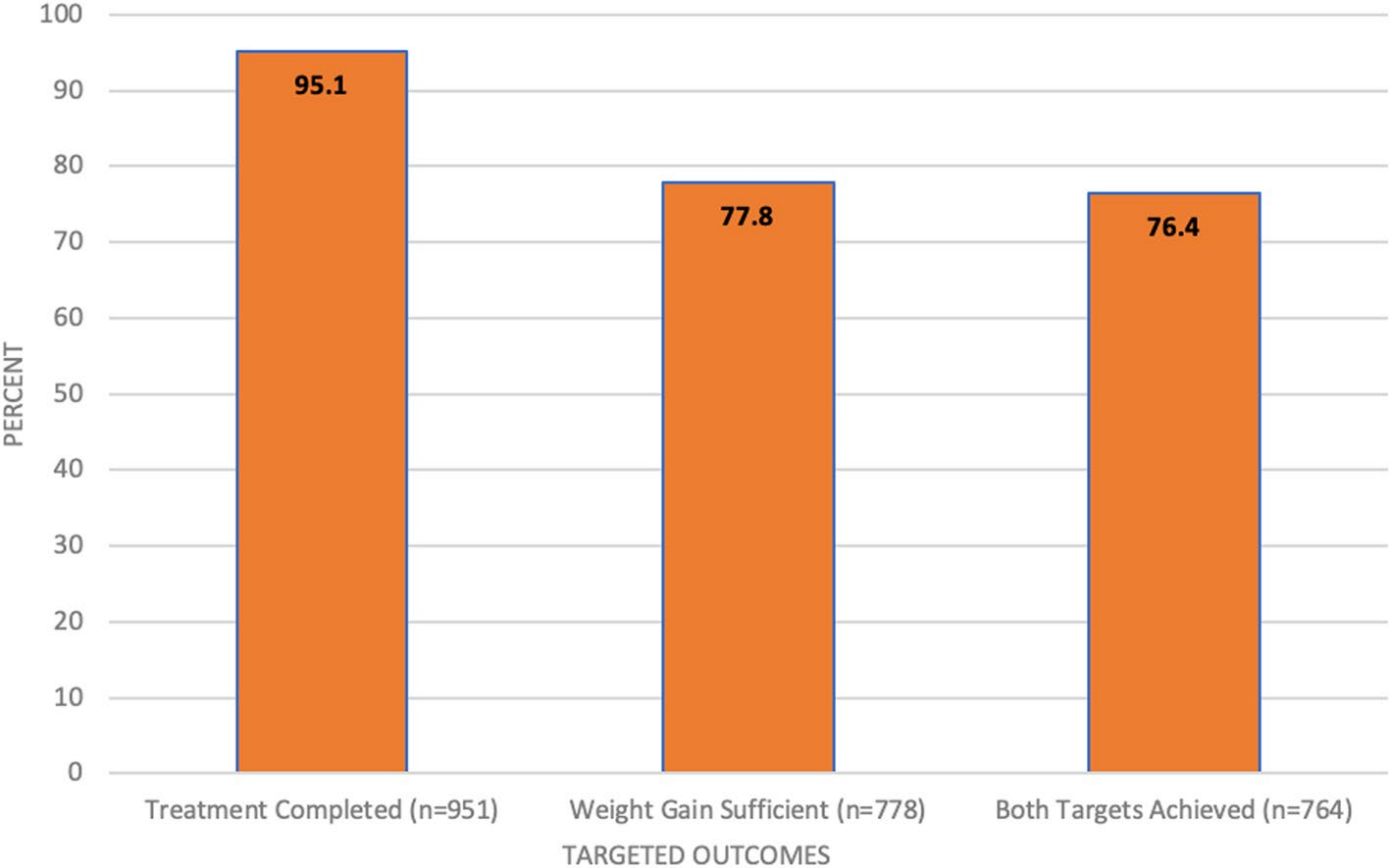
Program performance metrics

Figure 5 illustrates a final assessment of the 1000 phase 1 Mukti patients, according to whether they completed treatment and/or met the weight gain targets. Over 95% of patients met the treatment completion target. The weight gain target was achieved by 77.8% of patients. Overall, 76.4% of patients met both targets. Most of the patients who did not meet one or both targets were drop-outs or deaths. Many of these patients were lost early in the program, before the intervention could affect their outcomes.

### Cost of the Mukti program and the effect of results-based financing incentives

Table 9 shows the cost of the program during implementation of phase 1.

**Table 9:** Cost of Mukti services for phase 1

Cost category	Total cost (rupees)	Total cost (%) (US $)	Cost per patient (1000 patients) (US $)	Cost per patient reimbursed (764 patients) (US $)
Cluster coordinators and their direct supervisors	5,893,329	78,578 (50.2%)	79	103
Food baskets including delivery costs	3,118,402	41,579 (26.6%)	42	54
Technical costs (for example, for database, links to direct deposits, and technical monitoring)	718,476	9,580 (6.1%)	10	13
Start-up workshop	84,952	1,133 (0.7%)	1	1
Other direct costs, such as rent, utilities, and field travel	436,788	5,824 (3.7%)	6	8
Other (educational materials, PD sessions, weight machines)	420,491	5,607 (3.6%)	6	7
Administrative and management costs	1,067,244	14,230 (9.1%)	14	19
Grand total	11,739,682	156,529 (100.0%)	157	205

Tabulated costs represent per-patient marginal costs, not total program costs. The marginal cost for all phase 1 patients was 11,739,682 Indian rupees, or $156,529 U.S. dollars. Just over half of these costs covered the cost of hiring and supervising 15 cluster coordinators. An additional substantial expense was the cost of food baskets (26.6%). The average cost of the 6184 food baskets distributed was $6.72. The remainder of costs (about 20%) were for a miscellaneous set of overhead costs such as equipment, rent, and administration.

Table 9 also shows mean per-patient costs. The first is a per-patient cost for all 1000 Mukti patients which is $157, substantially lower than the negotiated per-patient reimbursement from USAID at $166. According to the final data submitted to USAID for payment, 764 of the 1000 Mukti patients met both the targeted outcomes (see Fig. 5 above). These are the patients for whom ChildFund received payment, which is slightly above the budgeted 75% who should meet both targets (as experienced in the pilot feasibility study). ChildFund thus—as negotiated in the RBF agreement—was reimbursed $126,824 (764 × $166). This compares to total costs of $156,529. The reason for the loss, is that the original budgeting did not take into consideration that costs would be incurred for the patients who died or dropped out early in the program. These patients did not receive a full package of services, and to a large extent their outcomes were beyond the control of the Mukti program. The reimbursement difference was made up by USAID, so that ChildFund could continue to expand the program.

While the RBF approach led to a loss for ChildFund, there were other positive self-reported results of the program as described by those interviewed. They said that the RBF system led to an increased focus on program outcomes by Mukti staff, along with improved accountability and transparency in program activities. This is consistent with other literature on RBF initiatives [44]. Even though they were not themselves incentivized to increase the rates of either weight gain or treatment completion, the staff all knew that reimbursement was driven by achieving the program goals. Another related positive result is that the government was engaged in the program through their role assessing program outcomes; this reportedly led to improvements in the Nikshay data system.

## Discussion

In March 2020, just as the COVID-19 lockdown began, ChildFund India began an innovative program to improve the nutrition of TB patients in the Dhar District of Madhya Pradesh with the support of USAID under its flagship PAHAL project. This evaluation has revealed several key findings concerning the program’s implementation, outcomes, and costs. In terms of service delivery, patients received regular home visits by cluster coordinators, more than one a month on average, and also received an average of six nutritious food baskets. In many cases, these food baskets were a major source of food for patients and their families during the COVID-19 lockdown. They attended, on average, more than two group meetings with instruction on nutrition, and most were helped to receive additional financial benefits from the government DBT system. This is substantially more assistance and education than is provided routinely to TB patients in Dhar District or other parts of Madhya Pradesh. According to patients in focus group discussions, the assistance was well regarded and helpful to them.

This research adds new evidence to the large body of literature on TB and nutrition, especially enhancing what is known about such programs during the COVID-19 lockdown in India. Data on weight gain came from program records and shows that most patients who remained with the program for some time (more than 2 months) did gain substantial weight (an average of 6.2 kg gained for adults). The analysis presented here leads to less clear conclusions concerning the program effect on treatment completion. This could be because completion rates were already high before Mukti began.

The RBF approach to reimbursing ChildFund is another important innovation. The evaluation examined whether and how that approach affected outcomes and costs. The costs to serve the patients ($157 per patient) were lower than the original estimate ($166). However, the amount reimbursed by USAID to ChildFund was initially less than program cost due to the results-based financing scheme. USAID made up the difference so that ChildFund would have funds to expand the program, considering that this was a demonstration program of relatively small scale. Looking towards long term sustainability as an RBF approach, it is necessary to reconsider what reasonable targets and their measurements should be (for example, reconsidering how to deal with deaths and loss to follow-up). As Mukti is expanded across Madhya Pradesh, it will be important to track whether the losses are continuing, or whether—as expected—economies of scale take hold and reverse the losses. Numerous other studies have shown that implementing results-based financing may not lead to cost savings [44–46]. The savings may accrue to the funder, but not to the provider of services, which was true for phase 1 of Mukti. When the provider is a non-governmental organization (NGO), a substantial loss could lead to the NGO’s inability to continue offering the program.

Although we were limited in studying the program impact on weight gain due the lack of data for a comparison group, results indicate that the multi-dimensional aspect of the intervention was important for weight gain success. For example, it is likely that the link between positive deviance nutritional education sessions and food basket distribution created an incentive for TB patients to attend the PD sessions. Previous studies showing the close association between malnutrition and TB [20–22] suggest that the extra food provided as part of Mukti, along with the nutrition counseling, likely improved TB patient nutritional status and consequently improved health overall. While patients reported changing their diets as a result of the food baskets and the educational sessions, they also said they often reverted to previous nutritional patterns after leaving the program, pointing to the need for more sustained forms of guidance.

Cluster coordinators, who made home visits and provided nutritional counseling, were also key to the success of the intervention. Without external donor funding, the future funding for this component is unclear. Currently, there are no home visits for TB treatment in Madhya Pradesh, and all services are provided at the DMCs. Undoubtedly, the fact that cluster coordinators visited patients at home created a much greater opportunity for one-on-one accessible nutritional education, especially during the COVID-19 pandemic. But, given the cost, alternative models should be explored with possibly a targeting of home-based services to those with the greatest need, as well as integration of nutritional education into the routine facility-based services.

### Limitations

The study has several limitations. First, the intervention took place under very unusual conditions at the beginning of the COVID-19 pandemic. It was also implemented in only one district of India, further limiting its generalizability. Transferring conclusions from this evaluation to another time or place should only be done after careful consideration of the context in which they were demonstrated.

Second, the weight gain data could only be collected for Mukti patients and not for an external comparison group. Thus, a rigorous impact analysis of this key outcome could not be performed for phase 1. An important next step in the assessment of the effectiveness of Mukti is a follow-up study comparing both outcomes—weight gain and treatment completion—to better understand the impact of Mukti. Such an evaluation is currently underway for phase 2 of Mukti, and these data results are forthcoming. Third, the data may have biases to an unknown degree. There may be inaccuracies, lags in reporting, incomplete data, or other deficiencies. The fact that we had only monthly aggregate data on treatment completion means that variability across patients within the month was not accounted for in the regression analysis.

Finally, there are many other potential positive contributions of the program which were not measured in the evaluation. These include potentially improved nutritional knowledge and practices, lower death rates, higher cure rates, reduction in reinfection, other measures of improved health status, and the potential for Mukti to have an impact on the overall public health delivery system. Since families gained new sources of food, the well-being of other family members may have improved and the learning from nutrition education could affect the family’s future nutrition. However, comments in the focus groups cast some doubt on whether nutrition practices were changed permanently from a relatively short-term intervention. These are all topics for future research into how to improve the nutritional status of TB patients.

### Strengths

A major strength of this evaluation is that it has both qualitative and quantitative components, which lends more credibility to findings. In addition, it draws on administrative data which provides a model for using existing data sets to study TB outcomes in India and other countries. Covering a long time period also controls for secular trends, such as the influence of COVID and changes in government programs and regulations. Finally, an excellent program database allowed for measuring the dosage of the program accurately.

## Conclusions

In sum, this research has provided findings that are pertinent to improving the nutrition of TB patients in Madhya Pradesh, India, and beyond using an RBF approach. The buy-in of multiple levels of government (national, state, and district), the donor (USAID), the implementation partner (ChildFund), and the knowledge manager (IPE Global) was critical to implementing this type of complex program. Further, the nutrition support apparently led to improved weight gain.

Future efforts, including in the expanded Mukti districts, should consider how to hold all stakeholders accountable, for example, treatment facilities and implementation staff, without unduly penalizing them for factors beyond their control such as early deaths and patients lost to follow-up. This suggests the possible need to incorporate a measure of patient illness severity in the payment scheme, as has been incorporated into some other RBF models. The evaluation of phase 2 of Mukti, which is underway, should examine the overall costs (including of donated staff time) more thoroughly, to determine the cost-effectiveness of Mukti and similar interventions.

## Supplementary Information

**Additional file 1.** Key Informant Interview Guide, Focus Group Discussion Guide, and Periods for Analysis of Treatment Completion.

## Data Availability

Data are not available due to confidentiality restrictions.

## Abbreviations

DBT: Direct benefits transfer
DMC: Designated microscopy center
DOTS: Directly observed treatment short-course
MDR-TB: Multi-drug resistant tuberculosis
P4P: Pay-for-performance
PAHAL: Partnerships for affordable healthcare access and longevity
PD: Positive deviance
PGIMER: Postgraduate Institute of medical education and research
RBF: Results-based financing
TB: Tuberculosis
USAID: United States agency for international development
