# Supplementary material for: Evaluation of a results-based financing nutrition intervention for tuberculosis patients in Madhya Pradesh, India, implemented during the COVID-19 pandemic
Source: BMC Glob Public Health. 2023 Sep 4;1:13. doi: 10.1186/s44263-023-00013-6 (PMC11622994; doi:10.1186/s44263-023-00013-6)
Supplement: Supplementary file 1 — Additional file 1. Key Informant Interview Guide, Focus Group Discussion Guide, and Periods for Analysis of Treatment Completion. [file 44263_2023_13_MOESM1_ESM.docx]

**Key Informant Interview Guide**

| **Question/Prompt** | **Purpose** |
| --- | --- |
| 1. Start with introductions and explain the purpose of the discussion. Explain what ChildFund’s interest is in SRH, particularly in the three countries most interested: Brazil, Guinea, and India. Refer to the lit. review write-up sent to them previously. | Build a rapport with the respondent, establish trust, ensure they have a firm understanding of the purpose of the exercise. |
| 1. To start: can you tell me a bit about your experience with implementing digital health interventions? For adolescents? For ASRH? In LMICs?  *FOLLOW UP PROMPTS:* 2. What types of projects have you worked on in the past? 3. What are your current digital-related projects, if any? 4. Have you conducted any evaluations on these projects (either published or unpublished)? If so, can you describe the results? 5. Either way, what types of evidence are you looking for in order to confidently advocate for digital delivery of health programming? | Establish baseline understanding of their history with the topic at hand. Obtain more details on their project history (types of projects, locations, etc.) and evaluation experience. |
| 1. From your perspective: do you consider digital health interventions to be a productive focus area for INGOs? Why or why not? | With their experience established above, we can ask them about their personal opinions on the subject. |
| 1. From your perspective, what are the most promising interventions so far (yours and those you know about), .Why do you think these are promising? We are particularly interested in knowing how many adolescents have been reached with your digital interventions. Which can be or have been scaled most effectively? Can you provide any reference materials?. We also would like to have any information on content/curricula. | Establish promising and scalable digital interventions from their perspective so we can cross-reference with our literature review. |
| 1. What do you believe are the main factors in the success of digital interventions?   *FOLLOW UP PROMPTS:*   1. What are the preferred platforms or technology solutions (e.g. WhatsApp, Facebook, etc.) in your perspective? 2. Based on your experience, are there appropriate priority populations? Ages, locations, etc. 3. What about the appropriate frequency of outreach? | Establish best practices from the perspective of the respondent. |
| 1. Do you know of any rigorous impact studies of digital interventions that are underway or have recently  been evaluated that are not on our list of references and included in our review?  If so, can you point us to those papers?j We are particularly interested in any cost or cost-effectiveness studies. | Identify any forthcoming impact evaluations that our literature review might have missed. |
| 1. Time To Develop Digital Intervention: Our country teams are struggling a bit trying to balance conflicting goals—finding the time to develop digital interventions versus the ambition to rapidly experiment and “fail fast.” In the interviewee’s experience, how long does it take to move from conceptualization to having a product to test? 2. Cost of Digital Intervention (not cost to develop intervention): Often, part of the rationale of digital interventions is that more people can be reached at a lower cost. In the interviewee’s experience, once the digital intervention is being implemented, is there a cost per participant that the intervention is targeting to be below in order to be “affordable” over the long term? 3. Team Skills/Competencies: In our case, country teams lead the digital intervention development process. These teams are usually composed primarily of program staff that specialize in the intervention (ASRH, social & emotional learning, prevention of violence in schools—these are examples). They do not have special training in, for example, human centered design, or deep knowledge of technology. 4. Any other information you would like to share with us? | We are looking for a rough estimate/range—a year, 2 years, 6 months, that kind of increment.    A rough estimate is what we’re looking for—like, the intervention should cost less than $4.00 or $2.00 or $8.00 /participant. If they have a reason for citing a target, that would be good to know (like it should be less expensive than a traditional, face to face intervention, where the cost should be under $X/participant.  These capabilities usually come later in the development process through a partnership with a tech company. In the experience of the interviewees, do they look to form teams with an array of skills, and if so, what do they competencies do they prioritize?  Probe for any additional information we might have missed that could be valuable. |
| 1. Thank the respondent for their time. Ask if it is okay to follow up with any further questions or if they would like to review the results of the discussion (assuming we can share). | Close the discussion and establish possibility of follow up discussions or contact. |

**Focus Group Discussion Guide**

| **Topic** | **FGD Guidelines** |
| --- | --- |
| Preamble | Thanks and welcome remarks Explain nature of the focus group (informal, multiway, participatory, disagree) Explain there are no right or wrong answers (all about finding out what people think) Explain about audio and video recording Tell about refreshments Explain the purpose of FGD: To talk about TB and Mukti interventions Ask for any questions or concerns? |
| Introduction | Ask all participants to introduce themselves one by one: Name, age, occupation, qualification Time since on treatment Current TB status |
| Knowledge | Discuss and probe the participants on the following: What causes TB? How does TB spread? Is TB life threatening?  What happens to the patient? Is TB curable without medicines? |
| Attitudes | Discuss and probe the participants on the following: Do you think TB should be taken seriously? Is it important to take medicines for all of 6 months? Are TB medicines difficult to consume?  Do you think TB medicines have any side effects? Do you know of somebody who recovered without taking treatment? |
| Practices | Discuss and probe the participants on the following: Did you get tested after 2 weeks of cough? What prompted you to go to the doctor? What convinced you to keep on taking medicines? Did you sincerely take all medicines as per regimen or some got skipped? Did you face any side effects due to medicines? |
| Socio-cultural practices | Discuss and probe the participants on the following: Do your family members know about your TB diagnosis and treatment? When did you tell your family about this? What was their reaction when they came to know? Do your friends and neighbours know about your TB diagnosis and treatment? What was their reaction when they came to know? Has anybody advised you to take alternative therapy/ or visit traditional healers/ shamans for TB treatment? Have you ever taken any alternative therapy/ or visited traditional healers/ shamans for TB treatment? Did getting afflicted with TB had any effect on your marriage? Did getting afflicted with TB had any effect on your job and your family’s financial situation? Did getting afflicted with TB had any effect on your or your child’s education? |
| Nutrition | Discuss and probe the participants on the following: Do you think diet plays any role in TB treatment?  Do you think healthy diet can cure TB? What is the impact of a good diet on TB patient’s health? Have your dietary practices changed during the last six months? How have they changed? Can you provide some examples? |
| Food baskets | Discuss and probe the participants on the following: Did you receive regular food baskets? Were the contents of the baskets sufficient for you for a month? Did you also share some of the food with other family members? Do you think the food baskets were helpful with your recovery? How can the baskets be further improved? |
| Counselling sessions | Discuss and probe the participants on the following:  Did you ever receive any counselling at home from a cluster coordinator? How many times do you think you were counselled? What was discussed by the cluster coordinator in these meetings? Did he discuss the importance of nutrition?  Do you think such meetings are helpful?  What else do you think should be done to make these meetings more beneficial? |
| Deviance sessions | Discuss and probe the participants on the following: Did you ever participate in a peer learning deviance session? How many times do you think you have participated? What was discussed in these meetings? Do you think such meetings are helpful? What else do you think should be done to make these meetings more beneficial? |
| Direct benefit cash transfer | Discuss and probe the participants on the following: Have you ever received any money from the Government? How many times have you received any amount? Is the money transferred monthly or on an irregular basis? Do you think the amount you receive is helpful? What do you utilize the money received for? Do you think the amount you receive is sufficient? |

**Periods for Analysis of Treatment Completion**

| **Cohort Period Number**  **(Number of Months of Initiation/Completion)** | **Type of Period** | **Initiation** | **Completion** |
| --- | --- | --- | --- |
| One  (12 months) | Mixed Period: Some pilot Mukti programming | May, 2018-April, 2019:  N=5018 (Dhar)  N=2192 (Jhabua) | November, 2018-October, 2019 |
| Two  (4 months) | Comparison Period: no Mukti programming in period | May-August, 2019:  N=1588 (Dhar)  N=798 (Jhabua) | November, 2019-February, 2020 |
| Three  (4 months) | Mixed Period: Some Phase 1 Mukti programming in period | September, 2019-January, 2020: N= 1769 (Dhar)  N=1248 (Jhabua) | March, 2020-July 2020 |
| Four  (3 months) | Covid Lock-Down Period: Limited Mukti programming | February-April, 2020:  N=869 (Dhar)  N=418 (Jhabua) | August-October, 2020 |
| Five  (4 months) | Treatment Period: Full Phase 1 Mukti programming | May-August, 2020:  N=979 (Dhar)  N=618: (Jhabua) | November, 2020-February, 2021 |
